# *Enterococcus faecium* NCIMB 10415 Modulates Epithelial Integrity, Heat Shock Protein, and Proinflammatory Cytokine Response in Intestinal Cells

**DOI:** 10.1155/2015/304149

**Published:** 2015-04-08

**Authors:** Shanti Klingspor, Angelika Bondzio, Holger Martens, Jörg R. Aschenbach, Katharina Bratz, Karsten Tedin, Ralf Einspanier, Ulrike Lodemann

**Affiliations:** ^1^Institute of Veterinary Physiology, Department of Veterinary Medicine, Freie Universität Berlin, Oertzenweg 19b, 14163 Berlin, Germany; ^2^Institute of Veterinary Biochemistry, Department of Veterinary Medicine, Freie Universität Berlin, Oertzenweg 19b, 14163 Berlin, Germany; ^3^Institute of Food Hygiene, Department of Veterinary Medicine, Freie Universität Berlin, Königsweg 69, 14163 Berlin, Germany; ^4^Institute of Microbiology and Epizootics, Department of Veterinary Medicine, Freie Universität Berlin, Robert-von-Ostertag-Straße 7-13, 14163 Berlin, Germany

## Abstract

Probiotics have shown positive effects on gastrointestinal diseases; they have barrier-modulating effects and change the inflammatory response towards pathogens in studies *in vitro*. The aim of this investigation has been to examine the response of intestinal epithelial cells to *Enterococcus faecium* NCIMB 10415 (*E. faecium*), a probiotic positively affecting diarrhea incidence in piglets, and two pathogenic *Escherichia coli* (*E. coli*) strains, with specific focus on the probiotic modulation of the response to the pathogenic challenge. Porcine (IPEC-J2) and human (Caco-2) intestinal cells were incubated without bacteria (control), with *E. faecium*, with enteropathogenic (EPEC) or enterotoxigenic *E. coli* (ETEC) each alone or in combination with *E. faecium*. The ETEC strain decreased transepithelial resistance (TER) and increased IL-8 mRNA and protein expression in both cell lines compared with control cells, an effect that could be prevented by pre- and coincubation with *E. faecium*. Similar effects were observed for the increased expression of heat shock protein 70 in Caco-2 cells. When the cells were challenged by the EPEC strain, no such pattern of changes could be observed. The reduced decrease in TER and the reduction of the proinflammatory and stress response of enterocytes following pathogenic challenge indicate the protective effect of the probiotic.

## 1. Introduction

Probiotic bacteria have been shown to have positive effects on hosts with intestinal diseases such as* Clostridium difficile*-, Rotavirus-, and* Escherichia coli*-induced diarrhea and also in the prevention of antibiotic-associated diarrhea and nosocomial infections [1–5]. According to the WHO, probiotics are defined as live organisms that, when ingested in sufficient amounts, have a beneficial effect on the overall health of the host [6]. In animal nutrition, probiotics are used as feed additives with positive effects regarding health and growth performance traits [7–9].

The probiotic* Enterococcus faecium* NCIMB 10415 (*E. faecium*) is licensed as a feed additive for sows and piglets and has been demonstrated to reduce diarrhea incidence and severity in weaning piglets [10, 11]. In human medicine,* E. faecium* is used successfully in the treatment of acute diarrheal diseases and in the prevention of antibiotic-associated diarrhea [12, 13]. However, the crosstalk between the probiotic and other microorganisms and between the probiotic and epithelial and immune cells of the intestinal wall is extremely complex [14, 15], and many of the underlying signaling mechanisms are still only partially understood.

*In vivo* models of gastrointestinal infections have demonstrated a positive effect of various probiotic feed additives on the functional barrier of the intestine [16–18]. These results are supported by data from* in vitro* cell culture infection models in which probiotic strains prevent or ameliorate damage to epithelial integrity by a pathogenic challenge [19, 20]. Furthermore, the immunological response of the mucosa can be influenced by probiotic strains, which modulate the release of cytokines, amongst other effects [21, 22]. For example, culture supernatant of* Lactobacillus plantarum* 2142 had a suppressive effect on the interleukin-8 (IL-8) and tumor necrosis factor-*α* (TNF-*α*) levels induced by oxidative stress in IPEC-J2 cells [23].

The expression of heat shock proteins (HSPs), a set of proteins that are involved in many regulatory pathways and that serve as chaperones for preserving cellular proteins, can be influenced by the intestinal microbiota and by probiotics [24–26]. Some of these HSPs, such as the inducible form of HSP70, are upregulated by several noxious conditions and can therefore be considered as cellular stress indicators [27]. HSPs and cytokines, in turn, are able to regulate barrier properties by influencing tight junction proteins and the structure and function of the cytoskeleton or transport properties of the epithelium [24, 28].

In previous studies, feed supplementation with* E. faecium* has been demonstrated to increase the absorptive and secretory capacity and improve barrier function of the small intestinal epithelium of piglets [29, 30]. Furthermore the proinflammatory cytokine IL-1*α*, which is expressed in higher amounts in Peyer's patches of* E. faecium-*treated piglets [31], increases chloride secretion in the small intestine of pigs [29].

The hypothesis of the current study is that intestinal epithelial cells play an important role in innate immune responses during enteric infections and that* E. faecium*, in turn, modulates the severity of enteric infections* via* the altered generation of proinflammatory cytokines and HSPs in intestinal cells. Thus, the aim of the present study has been to investigate the influence of the probiotic* E. faecium* and two different pathogenic* E. coli* strains on the HSP and proinflammatory cytokine responses and the epithelial integrity of two intestinal epithelial cell lines. We have further tested whether pre- and coincubation with* E. faecium* change the epithelial cell response to pathogenic* E. coli* strains.

## 2. Materials and Methods

### 2.1. Cells and Culture Conditions

The cell cultivation is described in detail in Lodemann et al. [32]. The human epithelial intestinal cell line from colorectal adenocarcinoma, Caco-2 (ATCC Catalog number HTB-37, ATCC, Manassas, USA; passages 37–45), was used as a model for the human small intestine. The porcine intestinal epithelial cell line (IPEC-J2; passages 73–79) was used as a model for the pig small intestine. This cell line was established from the jejunum of a newborn pig [33] and kindly provided by Professor Dr. Anthony Blikslager (North Carolina State University, USA). The cells routinely tested negative for* mycoplasma* contamination.

Cells for the experiments were allowed to differentiate for 14 days (IPEC-J2) or 21 days (Caco-2). On the day prior to experiments, the cells were fed with serum- and antibiotic-free media.

### 2.2. Bacterial Strains

Three different bacterial strains were used for the experiments: (1) the probiotic strain* Enterococcus faecium* NCIMB 10415 (cultivated from Cylactin, DSM, Heerlen, the Netherlands), (2) the enterotoxigenic* E. coli* IMT4818 (ETEC, isolated from a two-week-old piglet with enteritis, O149:K91:K88 (F4), and found to be positive for the presence of virulence genes est-1a, est-2 (genes coding for heat stable enterotoxins I and II) and elt-1a/b (gene coding for heat labile enterotoxin I) by the polymerase chain reaction (PCR)), and (3) the human enteropathogenic* E. coli* E2348/69 (EPEC, serotype O127:H6, positive for the eae gene coding for the* E. coli* attaching-effacing factor).

The* E. faecium* NCIMB 10415 strain was cultivated in brain-heart infusion (BHI) broth (OXOID GmbH, Wesel, Germany) and the* E. coli* strains in LB medium according to Miller, containing 10 g/L tryptone (OXOID GmbH, Wesel, Germany), 5 g/L yeast extract (OXOID GmbH, Wesel, Germany), and 10 g/L NaCl, at a pH of 7.0.

After overnight incubation of the cells at 37°C, subcultures of bacteria were grown for 3 to 4 h until mid-log phase and then centrifuged. Cell pellets were washed twice in phosphate-buffered saline (PBS, Biochrom, Berlin, Germany). The bacteria were resuspended in antibiotic- and serum-free Caco-2 or IPEC-J2 cell culture medium to reach a concentration of ≈10^8^ colony-forming units (CFU)/mL.

The optical density was measured to determine the concentration of bacterial cells. The measurement was confirmed by serial dilution on agar plates. The intestinal cells were infected with 10^6^ bacteria per cell culture insert (1.12 cm^2^) or per well (1.91 cm^2^), corresponding to a multiplicity of infection (MOI) of about 10 bacteria per seeded cell. The bacteria were added to the apical pole of the cells.

### 2.3. Experimental Setup and Procedure

**Figure 1:**
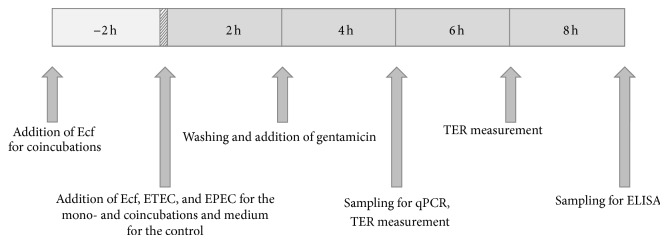
Timeline for the experimental setup.

For each experiment, the cell monolayers for the real-time quantitative PCRs (RT-qPCR), for enzyme-linked immunosorbent assay (ELISA), and for the transepithelial electrical resistance (TER) measurements were incubated for 2 h with the respective bacterial strains (ETEC, EPEC, or* E. faecium*) at a concentration of 10^6^ bacteria per cell culture insert or well (see Figure 1). The control cells received the equivalent amount of bacteria-free medium. Two hours after the bacterial incubation, the cells were washed twice with gentamicin-supplemented media to wash out and kill the bacteria, and gentamicin-supplemented media were added. Gentamicin was added to the media at a concentration of 50 *μ*g/mL (Biochrom, Berlin, Germany). The cells were incubated for various periods of time with regard to the different parameters measured, the exact time periods being given in Figure 1 under the specific headings. When the cells were incubated with the probiotic and either the ETEC or the EPEC together, the cells were preincubated with the* E. faecium* for 2 h, and then the pathogens were added. The cells were in contact with the pathogens for the same amount of time as in the monoincubation with the ETEC or EPEC. In the following, this experimental setup will be called “coincubation” and the incubation time will be given as the time that the cells were incubated with the pathogens.

A total of six independent experiments were performed for each cell line.

### 2.4. Transepithelial Electrical Resistance (TER) Measurements

For TER measurements, the cells were seeded at a density of 10^5^ cells on cell culture inserts (Transwell, clear polyester membrane, 12 mm diameter, 1.12 cm^2^ area, 0.4 *μ*m pore size; Corning B.V., Schiphol-Rijk, the Netherlands). The inserts for IPEC-J2 cells were coated with rat tail collagen type I (Serva Electrophoresis GmbH, Heidelberg, Germany). TER was measured by a Millicell-ERS (Electrical Resistance System; Millipore GmbH, Schwalbach, Germany). TER values were measured every two hours and corrected for the resistance of blank filters and for the membrane area.

### 2.5. Real-Time Quantitative Polymerase Chain Reaction (RT-qPCR)

For determining mRNA expression, the cells were seeded on 24-well cell culture plates (TPP, Biochrom, Berlin, Germany) at a density of 10^5^ cells/1.91 cm^2^. Confluent cell monolayers were incubated with the bacterial strains as described in the section “Experimental Setup.” After 4 h, cells were washed two times with PBS, stored in RNA*later* RNA Stabilization Reagent (Qiagen GmbH, Hilden, Germany), and frozen at −20°C.

The isolation of the total RNA of the harvested cells, the assessment of the RNA quality, and the cDNA synthesis are described in detail in other publications [29, 32]. The samples had to have a RNA integrity number above 7 to be used for RT-qPCR. For the synthesis of cDNA, 100 ng total RNA for the IPEC-J2 cells and 500 ng total RNA for the Caco-2 cells were used. Primer information is given in Table 1. Three reference genes were selected for normalization (ACTB, TBP, and UBP for the Caco-2 cells and GAPDH, TBP, and YWHAZ for the IPEC-J2 cells). The primer information for the reference genes can be found in Lodemann et al. [32].

RT-qPCR was performed and the relative amount of the target genes in the experimental groups was calculated by iQ5 software (Bio-Rad Laboratories GmbH, München, Germany) as described in Klingspor et al. [29].

### 2.6. Enzyme-Linked Immunosorbent Assay (ELISA)

For the ELISA experiments, the cells were seeded on 24-well cell culture plates (TPP, Biochrom, Berlin, Germany) at a density of 10^5^ cells/1.91 cm^2^, and incubation with bacterial strains was as described above. After 8 h, the supernatants of the cells were harvested, and species-specific IL-8 ELISAs were performed on cell culture supernatants according to the manufacturers' instructions (Invitrogen ELISA Kit, Swine IL-8, Invitrogen Life Technologies GmbH, Darmstadt, Germany, for IPEC-J2 supernatants; Thermo scientific human IL-8 ELISA Kit, Pierce Biotechnology, Rockford, USA, for Caco-2 supernatants). Assays were performed in duplicate.

For the HSP70 ELISA, protein was extracted from the cells. For this purpose, the Complete Lysis-M reagent set (Roche Diagnostics Deutschland GmbH Mannheim, Germany) was used, and the lysis buffer containing a mild detergent in bicine buffer and protease inhibitors was prepared following the manufacturers' instructions. The cells were washed with PBS, and 200 *μ*L lysis buffer was added to each well. After 5 min of incubation at room temperature and gentle shaking, the cell lysate was collected. HSP70 ELISA of the extracted protein was performed according to the manufacturers' instructions (Porcine HSP70 ELISA Kit, Bio-Medical Assay for IPEC-J2 cell extracts and HSP70 ELISA Kit, StressMARQ Biosciences for Caco-2 cell extracts). Assays were performed in duplicate.

### 2.7. Statistical Analysis

Statistical evaluations were carried out by means of the IBM SPSS-Statistics program for Windows, version 22 (International Business Machines Corp., Armonk, United States of America). Graphs were plotted with SPSS and Microsoft Excel 2010. Results are given as means ± SEM. *N* refers to the number of experiments.

Statistical significance of differences was assessed by variance analysis. The fixed factor was “treatment of the cells” (incubation with medium (control),* E. faecium*, ETEC, EPEC, ETEC or EPEC in coincubation with* E. faecium*). An overall analysis of the data for each cell line and each parameter (TER, mRNA-, and protein expression) was performed. A *P* value of <0.05 was assumed to indicate statistically significant differences. If a statistical significant difference occurred in the overall analysis each time point (4 h, 6 h, 8 h) was analyzed separately. In the case of a significant difference between groups (treatment of the cells), the Fisher least significant difference post hoc test was performed.

## 3. Results

### 3.1. TER Measurements

The TER values of confluent cell monolayers were recorded as a measure of epithelial integrity.

#### 3.1.1. Caco-2

**Figure 2:**
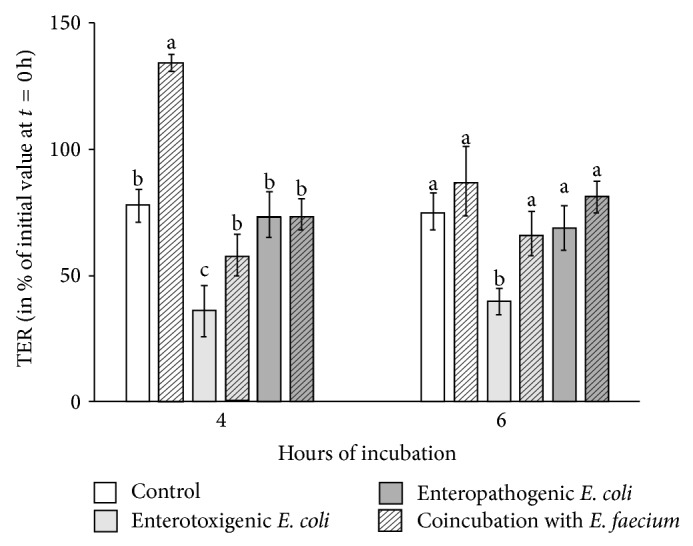
TER values of Caco-2 cells in percent of the initial value before the beginning of the experiment (*t* = 0 hours) (means ± SEM). Cell monolayers were treated with various bacterial strains for 4 h and 6 h. *N* = 6 independent experiments; different letters indicate significant differences between the differently treated cells (*P* ≤ 0.05).

The overall analysis revealed significant differences of TER between incubations with the various bacteria (*P* < 0.001). Monoincubation of the cells with the probiotic* E. faecium* caused an increase of TER compared with the control (at 4 h; Figure 2) indicating a positive effect of* E. faecium* on barrier function. Exposure of the cells to ETEC significantly decreased TER (at 4 h and 6 h), whereas the TER of EPEC-treated cells did not show significant alterations compared with the control. Cell monolayers coincubated with* E. faecium* and ETEC showed a significantly higher TER compared with cells incubated with ETEC alone.

#### 3.1.2. IPEC-J2

**Figure 3:**
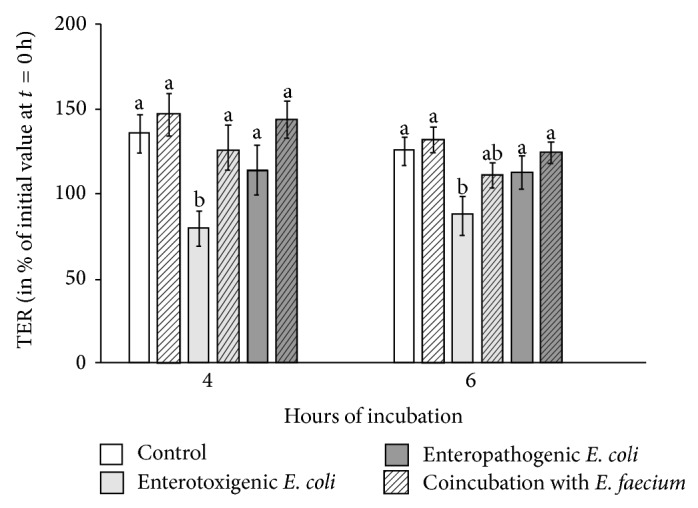
TER values of the IPEC-J2 cells in percent of the initial value before the beginning of the experiment (*t* = 0 hours) (means ± SEM). Cell monolayers were treated with various bacterial strains for 4 h and 6 h. *N* = 6 independent experiments; different letters indicate significant differences between the differently treated cells (*P* ≤ 0.05).

In IPEC-J2 cells, significant differences between the various bacterial incubations could also be detected (*P* = 0.01). In this cell line, the TER values were not affected in cells that were incubated with* E. faecium* alone compared with the control (Figure 3). However, the ETEC strain, again, caused a significant decrease in TER (4 h, 6 h), whereas the TER of cells incubated with EPEC did not differ significantly from the control. Pre- and coincubation with* E. faecium* reversed the decreased TER of ETEC-treated IPEC-J2 cell monolayers at 4 h and, as a trend, at 6 h, while TER of EPEC-treated cells was not changed by pre- and coincubation with* E. faecium*.

### 3.2. HSP Expression

As a stress indicator for the pathogenic challenge, HSP70 expression was tested in both cell lines at the mRNA and protein levels.

#### 3.2.1. Caco-2

In the overall analysis, the differences between the treatment groups were statistically significant for the mRNA expression of HSP70 (*P* = 0.001).

**Figure 4:**
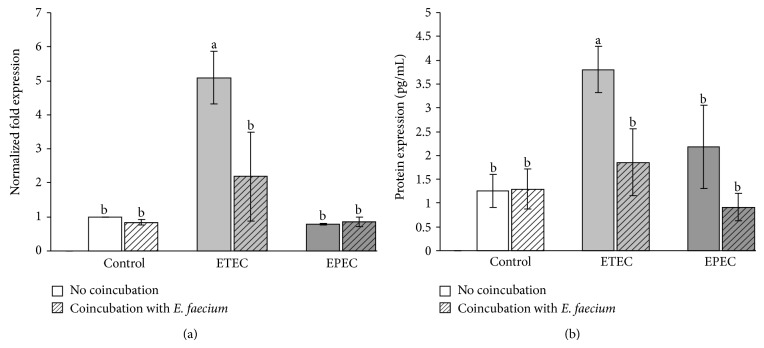
HSP70 expression in Caco-2 cells after treatment with various bacterial strains (means ± SEM). (a) mRNA expression, *N* = 5 independent experiments and (b) protein expression, *N* = 4 independent experiments; different letters indicate significant differences between the differently treated cells (*P* ≤ 0.05).

After four hours of incubation, no differences could be observed between control cells and cells incubated with EPEC or* E. faecium* (alone or in combination with ETEC and EPEC) (Figure 4(a)). However, the mRNA expression of HSP70 was significantly higher in the cells that were incubated with the ETEC strain alone compared with all other treatments. This implied that the coincubation of ETEC with* E. faecium* significantly reduced the increase in mRNA expression observed during incubation with ETEC alone.

These results were reflected by the changes in HSP70 protein expression (Figure 4(b)).

#### 3.2.2. IPEC-J2

**Figure 5:**
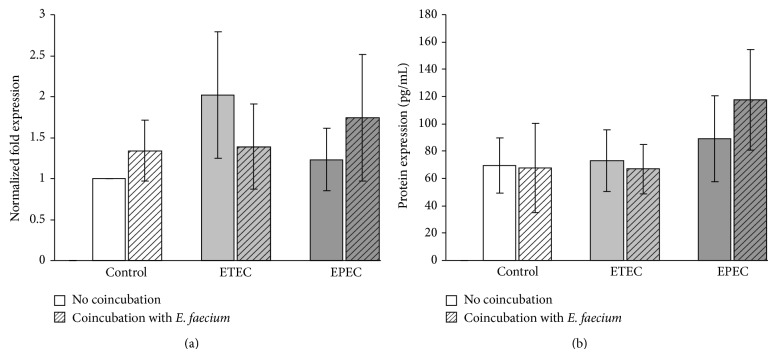
HSP70 expression in IPEC-J2 cells after treatment with various bacterial strains (means ± SEM). (a) mRNA expression, *N* = 6 independent experiments and (b) protein expression, *N* = 4 independent experiments.

For IPEC-J2 cells, the mRNA and protein expression of HSP70 showed some numerical, but no statistically significant, differences between the treatment groups (Figure 5).

### 3.3. IL-8 Expression

The cytokine IL-8 was chosen as a measure of the proinflammatory response of the cells.

#### 3.3.1. Caco-2

**Figure 6:**
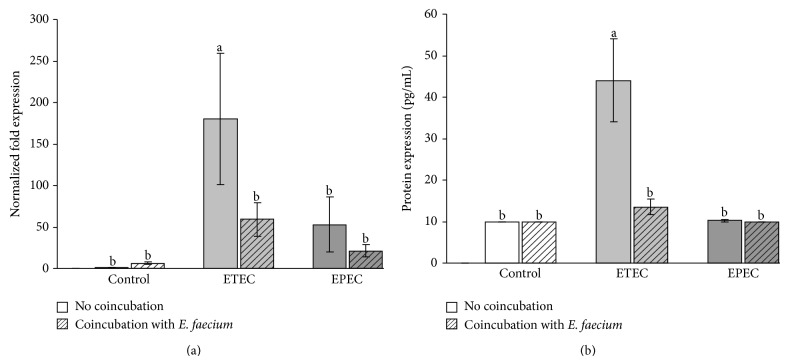
IL-8 expression in Caco-2 cells after treatment with various bacterial strains (means ± SEM). (a) mRNA expression, *N* = 5 independent experiments and (b) protein expression, *N* = 5 independent experiments, different letters indicate significant differences between the differently treated cells (*P* ≤ 0.05).

The bacterial incubation significantly affected the mRNA expression of IL-8 (*P* = 0.008). The mRNA expression of IL-8 did not differ between the cells incubated with* E. faecium* and the control cells. However, it was significantly increased 4 h after incubation with the ETEC strain compared with the control and the* E. faecium-*treated cells. Coincubation with* E. faecium* significantly reduced this increase (Figure 6(a)). Although a numerical increase was seen in all groups incubated with bacteria, the mRNA expression did not differ significantly between the other treatment groups and the control (Figure 6(a)).

The changes in mRNA expression were mirrored by similar changes in IL-8 protein expression (Figure 6(b)).

#### 3.3.2. IPEC-J2

**Figure 7:**
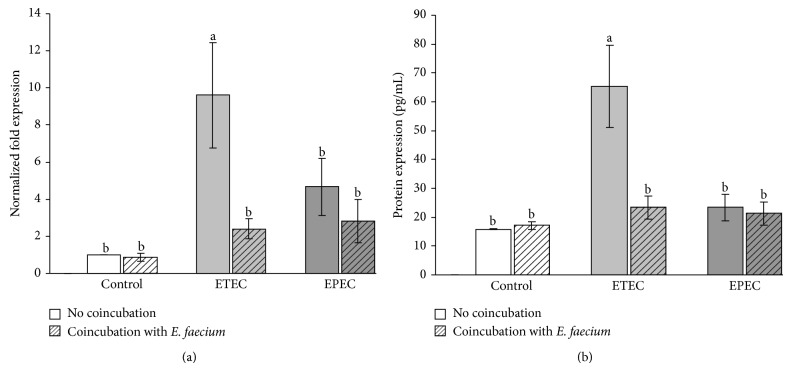
IL-8 expression in IPEC-J2 cells after treatment with various bacterial strains (means ± SEM). (a) mRNA expression, *N* = 6 independent experiments and (b) protein expression, *N* = 6 independent experiments; different letters indicate significant differences between the differently treated cells (*P* ≤ 0.05).

The results regarding IL-8 expression by the IPEC-J2 cell line were largely comparable with the results obtained for Caco-2 cells. In the overall analysis, significant differences for IL-8 mRNA (*P* = 0.001) and protein expression (*P* = 0.001) were observed, and statistical differences between individual groups were as described for Caco-2 cells (Figure 7).

## 4. Discussion

The aim of this study was to elucidate the effects of the probiotic* E. faecium* on barrier function and the inflammatory response of the intestinal epithelium, the latter effect being, in addition to other functions, an important part of the immune system of the gut. To examine whether the probiotic strain could modify the epithelial response to a pathogenic challenge, epithelial cell monolayers were incubated with two selected pathogenic* E. coli* strains. Our hypothesis was that epithelial integrity might be improved, whereas the expression of HSP70 and proinflammatory cytokines might be reduced because of the action of* E. faecium*.

Such “challenge” experiments can be carried out in various ways. One approach is to treat the confluent cell monolayers directly with the pathogenic bacteria for the whole duration of the experiment. The second is to incubate the epithelial cells with supernatants of the bacterial cultures [23, 38]. The advantage of using supernatants is the avoidance of pH decreases in the medium or nutrient competition by the rapidly growing bacteria [39]. However, supernatants or dead bacteria cannot mimic the conditions of living bacteria and exclude potential direct interactions of living bacteria with the epithelial cells. To include such bacteria-epithelial interactions, the experimental design of the present study included an initial incubation with living bacteria for 2 h. Thereafter, bacteria were killed by gentamicin, with the intention of diminishing secondary effects of the bacterial incubation. Two intestinal epithelial cell lines were used: porcine IPEC-J2 and human Caco-2 cells.

The Caco-2 cell line is well established and has been used for many years to investigate specific aspects of small intestinal function, including investigations on the effects of probiotic bacteria [40, 41]. Nonetheless, it has limitations because of its cancerogenic origin from the colon. The porcine IPEC-J2 cells seem to be a suitable model to mimic* in vivo* conditions of the small intestine, as they have no tumorous origin and were originally isolated from the small intestine [30, 42]; they have been recently used to study other probiotic microorganisms [23, 39]. The second aim of this study has therefore been to compare Caco-2 and IPEC-J2 cells under the same experimental conditions to provide further evidence for the use of IPEC-J2 cells as a model for small intestinal simulation.

### 4.1. TER

TER is a compound measure of paracellular and transcellular resistance and has been used to assess epithelial integrity in probiotic studies [43, 44]. In the present study, the TER of both cell lines was slightly increased after 4 h of incubation with* E. faecium* alone, but significantly only in Caco-2 cells. These results are in agreement with data of earlier studies showing that various probiotic bacterial strains had either no or an enhancing effect on the TER of intestinal epithelial cells of various origins [45–47]. In porcine IPEC-J2 cells, no enhancing effect has been reported so far [23]. The ETEC strain but not the EPEC strain significantly reduced TER in both cell lines compared with the control; this indicates a change in the epithelial integrity as shown previously by other authors [48, 49]. This decrease was inhibited when cell monolayers were (pre- and) coincubated with the* E. faecium* strain. Similar results have been obtained for other probiotic strains in* in vitro* infection studies with various intestinal cell lines of human origin, such as T84, HT29, or Caco-2 cells [43, 44, 50]. In a porcine cell model (IPEC-1)* Lactobacillus sobrius* DSM 16698 also inhibited the decrease of TER caused by an ETEC strain [51].

In former studies, a probiotic action to prevent the TER decrease induced by ETEC has been correlated with the inhibition of* E. coli* adhesion, which is the first step of ETEC infection [52]. Another possibility by which* E. faecium* could prevent the decrease of TER after ETEC infection might be related to tight junction (TJ) protein expression and localization or to the cytoskeletal organization [51].

These parameters have not been assessed here but will be the target of further studies.

### 4.2. HSP70

HSPs protect cells, tissues, and organs against various types of stress factors by reducing or avoiding the stress-induced denaturation of proteins. Gene expression of inducible HSP is triggered by many different stressors and is mainly regulated at the transcriptional level [53–55]. In the gastrointestinal tract, the expression of inducible HSP is markedly influenced by the intestinal microbiota [56, 57].

The inducible form of HSP70, which was assessed in the present study, is often considered to be cytoprotective as its induction by minor stressors can protect the tissue from major challenges [58, 59]. Some probiotic bacteria obviously activate this cytoprotective mechanism as part of their mode of action [23, 60, 61].* E. faecium* alone, however, did not alter expression of HSP70 in the present study, indicating that different probiotics rely on different mechanisms and pathways to exert their effects on the host [15, 60].

However, in the Caco-2 cells, the expression of HSP70 was significantly increased in the cells incubated with the ETEC. This increase could be prevented by probiotic pre- and coincubation. In the IPEC-J2 cells, a similar tendency could be observed.

Although, as stated above, the upregulation of HSP70 is often interpreted as a positive effect because of its cytoprotective effects, evidence has also been presented suggesting that HSP70 expression is an indicator of pathological stress [27].

In conjunction with the effects on TER and the proinflammatory cytokine response, the increased expression of HSP70 after pathogenic challenge indicates a stress reaction of the intestinal cells. As such, the reduced increase in HSP70 after pre- and coincubation with* E. faecium* in the present study suggests that cells are less damaged by ETEC.

### 4.3. IL-8

The proinflammatory cytokine response in the gastrointestinal tract is influenced by the gut microbiota. Among others, the cytokine expression of epithelial cells can be modulated by probiotic strains [21, 40, 62]. Altered cytokine release, in turn, can regulate the structure and function of TJ and cytoskeleton [63, 64] and the transport properties of epithelial cells [65, 66].

In the present study, IL-8 has been chosen as a representative cytokine of the epithelial proinflammatory response. IL-8 belongs to the proinflammatory “chemokine” family and is reported to induce neutrophil and T-lymphocyte chemotaxis, neutrophil activation, and the enhanced expression of neutrophil adhesion molecules [67, 68]. One of the responses of intestinal epithelial cells as part of the innate immune system to various inflammatory stimuli is the production of IL-8 [69].

In the present study, IL-8 expression was considerably increased when the cells were incubated with ETEC; this increase was abrogated by concomitant incubation with* E. faecium*. Similar reductions of proinflammatory responses to a pathogen by probiotic strains have been observed in other* in vivo* and* in vitro* models [23, 41, 51, 70]. In some cases, this was accompanied by the maintenance of epithelial barrier function [70]. In porcine intestinal epithelial cells, various* lactobacilli* and bacilli strains counteract the increase in IL-8 and other proinflammatory cytokines elicited by stimulation with ETEC,* S. typhimurium*, oxidative stress, or lipopolysaccharide. In some cases, this is associated with the protection of the epithelial barrier [23, 39, 71].

Although IL-8 secretion is part of the innate immune response aimed at the elimination of pathogens, the persistent production of IL-8 accompanied by the constant infiltration of neutrophils leads to massive epithelial cell damage [72], which is one cause of diarrhea. Several studies have demonstrated that epithelial damage can be prevented by interventions that suppress the IL-8 levels in IBD [73, 74]. This could also be an approach for reducing epithelial damage and diarrhea in acute infections such as ETEC and, here, we consider it to be one of the positive effects of healthy microbiota [61]. Taking this into account, the effects of* E. faecium* observed in the present study indicate a protective effect of this probiotic in acute intestinal inflammation induced by ETEC.

The exact mechanisms by which* E. faecium* exerts its influence on cytokine secretion have to be further investigated.

## 5. Conclusion

Preincubation with the probiotic* E. faecium* abrogates or reduces all examined effects induced by the ETEC such as the HSP70 stress response, the elevated expression of the proinflammatory cytokine IL-8, and the decrease in TER as a measure of epithelial integrity.

A key feature of the intestinal immune system is its ability to protect against pathogens while avoiding a destructive inflammatory response. An exaggerated proinflammatory cytokine secretion such as IL-8 leads to disease states and, in this case, a reduction of proinflammatory cytokines together with the reduction of HSP70 expression and the prevention of potential epithelial damage might alleviate symptoms, indicating a positive effect by the probiotic. The underlying mechanisms will be the subject of further studies.

Both cell lines react in a similar manner to incubation with pathogens and the probiotic. Therefore, the IPEC-J2 cell line can be considered as a reliable model for studying the effects of probiotics on the protection of the intestinal epithelium from stressful conditions and inflammation. Furthermore, the parallel response in the two cell lines underlines the general transferability of the effects of* E. faecium* seen in the present study.

## Figures and Tables

**Table 1:** Oligonucleotide primers and amplicon length of PCR products.

Gene information	Primer sequence	Amplicon length	Accession number	Reference
*HSP70* (heat shock protein 70, *Homo sapiens*)	(S) 5′-ACT GCC CTG ATC AAG CGC-3′ (AS) 5′-CGG GTT GGT TGT CGG AGT AG-3′	81 bp	NM_005346	[34]

*IL8 *(*CXCL8*) (interleukin-8 (chemokine (C-X-C motif) ligand 8), *Homo sapiens*)	(S) 5′-ATG ACT TCC AAG CTG GC-3′ (AS) 5′-ACT TCT CCA CAA CCC T-3′	274 bp	NM_000584.3	[35]

*HSP70* (heat shock protein 70,* Sus scrofa) *	(S) 5′-GTG GCT CTA CCC GCA TCC C-3′ (AS) 5′-GCA CAG CAG CAC CAT AGG C-3′	114 bp	M29506	[36]

*IL8* (*CXCL8*) (interleukin-8, *Sus scrofa*)	(S) 5′-GGC AGT TTT CCT GCT TTC T-3′ (AS) 5′-CAG TGG GGT CCA CTC TCA AT-3′	154 bp	X61151	[37]
